# Reference range of fetal thorax using two-dimensional and three-dimensional ultrasound VOCAL technique and application in fetal thoracic malformations

**DOI:** 10.1186/s12880-021-00548-w

**Published:** 2021-02-22

**Authors:** Xihua Lian, Zhenhong Xu, Liping Zheng, Zhixing Zhu, Tofunmi Ejiwale, Ayush Kumar, Peiya Cai, Shaozheng He, Shunlan Liu, Ying Zhang, Guorong Lyu

**Affiliations:** 1 Department of Ultrasound Medicine, Second Affiliated Hospital of Fujian Medical University, No. 2 Ji’an Road, Fengze District, Quanzhou, China; 2 Collaborative Innovation Center for Maternal and Infant Health Service Application Technology of Education Ministry, Quanzhou Medical College, Quanzhou, China; 3 Department of Ultrasound Medicine, Second Affiliated Hospital of Xiamen Medical College, Xiamen, China; 4 Department of Respiratory and Critical Care Medicine, Second Affiliated Hospital of Fujian Medical University, Quanzhou, China; 5 Department of Pathology and Biomedical Science, University of Otago, Christchurch, New Zealand; 6 Department of Obstetrics and Gynecology, Second Affiliated Hospital of Fujian Medical University, Quanzhou, China

**Keywords:** Fetal thorax, 3D ultrasound, VOCAL, Reference range, Malformation

## Abstract

**Background:**

To establish the normal reference range of fetal thorax by two-dimensional (2D) and three-dimensional (3D) ultrasound VOCAL technique and evaluate the application in diagnosing fetal thoracic malformations.

**Methods:**

A prospective cross-sectional study was undertaken involving 1077 women who have a normal singleton pregnancy at 13–40 weeks gestational age (GA). 2D ultrasound and 3D ultrasound VOCAL technique were utilized to assess fetal thoracic transverse diameter, thoracic anteroposterior diameter, thoracic circumference, thoracic area, lung volume, thoracic volume and lung-to-thoracic volume ratio. The nomograms of 2D and 3D fetal thoracic measurements were created to GA. 50 cases were randomly selected to calculate intra- and inter-observer reliability and agreement. In addition, the case groups including congenital skeletal dysplasia (SD) (15), congenital diaphragmatic hernia (CDH) (30), pulmonary sequestration (PS) (25) and congenital cystic adenomatoid malformation (CCAM) (36) were assessed by the nomograms and followed up subsequently.

**Results:**

Both 2D and 3D fetal thoracic parameters increased with GA using a quadratic regression equation. The intra- and inter-observer reliability and agreement of each thoracic parameter were excellent. 2D fetal thoracic parameters could initially evaluate the fetal thoracic development and diagnose the skeletal thoracic deformity, and lung volume, thoracic volume and lung-to-thorax volume ratio were practical to diagnose and differentiate CDH, PS and CCAM.

**Conclusion:**

We have established the normal fetal thoracic reference range at 13–40 weeks, which has a high value in diagnosing congenital thoracic malformations.

## Background

The normal development of the thoracic structure is an essential basis for neonatal spontaneous breathing during the embryonic and fetal period, so the prenatal diagnosis of the fetal thoracic structure and its deformities is critical. Fetal congenital thoracic malformations (CTM) are diverse, such as congenital skeletal dysplasia (SD), congenital diaphragmatic hernia (CDH), pulmonary sequestration (PS) and congenital cystic adenomatoid malformation (CCAM) [1]. They can cause various complications, the most serious of which is pulmonary hypoplasia (PH). PH refers to a disease whereby the fetal lung is defectively developed or stunted during the fetal development process. This typically manifest via a reduction number of pulmonary cells, airways and alveoli, thereby reducing lung volume and weight. PH affects fetal lung gas exchange and is responsible for high fetal and neonatal morbidity and mortality [2, 3]. Congenital thoracic dysplasia is one of the causes of PH, as abnormal development of the thorax directly affects or restricts the lung development and accompanied by serious consequences [4]. Thus, early prenatal diagnosis of CTM is beneficial for timely pregnancy management in fetuses with deadly deformities [5]. However, few methods are currently available for evaluating fetal thoracic development internationally [1, 5]. In addition, there are only a few studies focusing on partial thoracic parameters reference ranges, such as fetal thoracic volume [6–8], and there are rare studies regarding the differential diagnosis of abnormal fetal thorax diseases. Therefore, it is imperative to determine a new and dependable method to evaluate the fetal thorax and establish a nomogram of thoracic parameters. Our study aims to: (1) combine two-dimensional (2D) ultrasound and three-dimensional (3D) ultrasound Virtual Organ Computer-aided Analysis (VOCAL) technique to evaluate the normal development of fetal thorax; (2) measure the fetal thoracic transverse and anteroposterior diameter, thoracic circumference, thoracic area, lung volume, thoracic volume and lung-to-thoracic volume ratio, and establish a normal reference range of various measurements; (3) further explore its application in the diagnosis of congenital SD, CDH, PS and CCAM.

## Methods

### Sample and protocol

This is a prospective cross-sectional study undertaken from 1 July 2014 to 1 July 2019. Pregnant women in the normal group and the abnormal groups were randomly selected and recruited into this study.

The inclusion criteria for the normal group included (1) singleton pregnancy, (2) precise gestational age (GA) based on last menstrual period and evaluated via ultrasonography before 20 gestational week, (3) GA is between 13 and 40 weeks, (4) absence of any fetal malformations, and (5) low-risk pregnancy without other maternal or placental complications. Exclusion criteria included (1) multifetal pregnancy, (2) any fetal malformations, (3) poor ultrasound imaging.

Abnormal group: All cases were confirmed by postpartum examination or autopsy.

To establish the fetal thoracic nomograms, we took measurements from a total of 1077 singleton and healthy pregnant women who met all above inclusion and exclusion criteria. The mean age of them was 27.40 years, the mean GA was 26.35 weeks. Meanwhile, 15 SD fetuses, 30 CDH fetuses, 25 PS fetuses and 36 CCAM fetuses were randomly selected, the mean GA was 19.89 weeks, 25.06 weeks, 25.42 weeks, and 25.84 weeks, respectively.

In addition, 50 normal fetuses were randomly selected to analyze the intra- and inter-observer reliability and agreement. The same investigator (X.H.) performed all the thoracic measurements twice to estimate the intra-observer reliability and agreement. Simultaneously, another sonographer (S.L.) conducted an extra measurement to determine the inter-observer reliability and agreement. Both examiners worked independently and were shielded from each other.

### Measurements

All ultrasound parameters were measured by GE E8 or E10 Expert device (General Electric Healthcare, Milwaukee, MI, USA) provided with a 4–8 MHz abdominal curvilinear transducer.

A routine standard obstetric ultrasound examination was performed for each fetus to determine the fetal morphology and biometry. To obtain a best acoustic window of the thorax, we scanned fetal thorax on the heart four-chamber view section. From this section, we obtained the fetal thoracic transverse and anteroposterior diameter, thoracic circumference, thoracic area, lung volume and thoracic volume. For the optimization of 3D volume acquisition, we standardized the opening scanning angle between 45° and 85°. The low speed, high quality and harmonic mode was selected respectively. The pregnant women were required to hold their breath for a short time when the fetus was motionless, then we activated the automatic scanning window to involve the entire fetal thorax. All images were saved in the machine and analyzed off line.

**Fig. 1:**
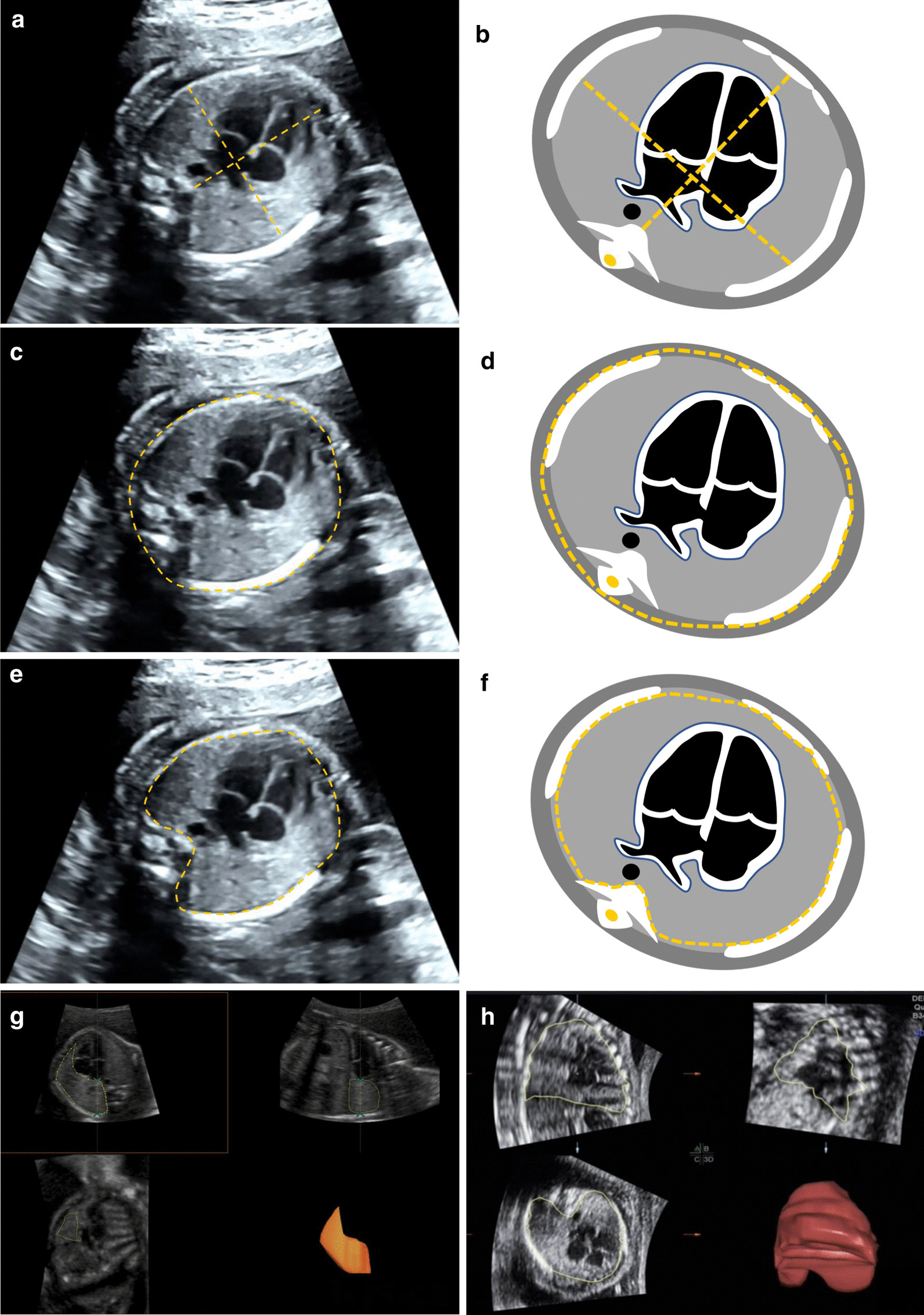
Ultrasonography and schematic diagram of fetal thoracic measurements. **a**, **b** Thoracic transverse diameter and thoracic anteroposterior diameter; **c**, **d** Thoracic circumference; **e**, **f** Thoracic area; **g** lung volume; **h** Thoracic volume

The distance between spinal front edge and sternum rear edge was the thoracic anteroposterior diameter; a straight line which was drawn perpendicular to the anteroposterior diameter and between the two thoracic inner edges was the thoracic transverse diameter (Fig. 1a, b). A circle was manually traced along the outer edges of the ribs, sternum, and spine to measure the thoracic circumference (Fig. 1c, d). Similarly, the thoracic area was the circle area that was manually traced along the inner edges of the ribs, sternum, and spine (Fig. 1e, f). 3D lung volume and thoracic volume were measured on the three perpendicular planes, VOCAL software (General Electric Medical Systems, KretzTechnik) was used by delimitating the surface with a rotation angle of 15° (12 planes) to acquire the volume automatically on the plane A. Briefly, in terms of lung volume measurement, we drew the lung outline excluding heart, organs in the mediastina, ribs and spine on each rotation plane 12 times. Left and right lung was measured separately, and added together to calculate the overall lung volume. To obtain the thoracic volume, we rotated the z-axis to make sure that the lung apex was above and the diaphragm was below on plane A. The thoracic contour (entire inner margin of thorax and upper margin of the diaphragm) was defined on each plane. After contouring the last plane, the reconstructed lung and thorax 3D images were established (Fig. 1g, h).

### Statistical analysis

All data were analyzed by SPSS software (version 21.0, IBM Corp., Armonk, NY, USA) and Medcalc software (Mariakerke, Belgium). Continuous variables were stated as mean and standard deviation (SD). We used the quadratic regression model as the best equation for evaluating correlation between each thoracic parameter and GA. Coefficient of determination (R^2^) was used to calculate the adjustments. According to the best-fit equation, predictive values for mean, SD, 2.5th, 50th, and 97.5th percentile ranges of each fetal thoracic parameter were constructed between 13 and 40 weeks. As all thoracic parameters increase with increasing GA, Z score was used to eliminate the effects of GA when comparing the measurements between the abnormal and normal groups. Z score = (measured thoracic value − overall mean thoracic value)/overall standard deviation of thoracic value. The *Mann–Whitney U* test was performed to compare the data between the abnormal and normal groups. We applied intraclass correlation coefficient (ICC) to calculate the reliability and performed Bland–Altman plots to assess agreement via showing bias between the two values and the limits of agreement (LoA) [9]. The reliability quality could be interpreted excellent if the ICC cutoff value was more than 0.90 [10]. All tests were considered significant with *p* < 0.05.

## Results

### Fetal thoracic identification rate and normal ultrasonography

There were 1167 pregnant women selected in our study. Of these, 90 fetal images which were affected by thick abdominal fat in pregnant women (n = 25), attenuation of fetal rib (n = 28), fetal position (n = 20) and amniotic fluid volume (n = 17) were excluded. The remaining 1077 women were included in this study, so the identification rate is 92.29%.

**Fig. 2:**
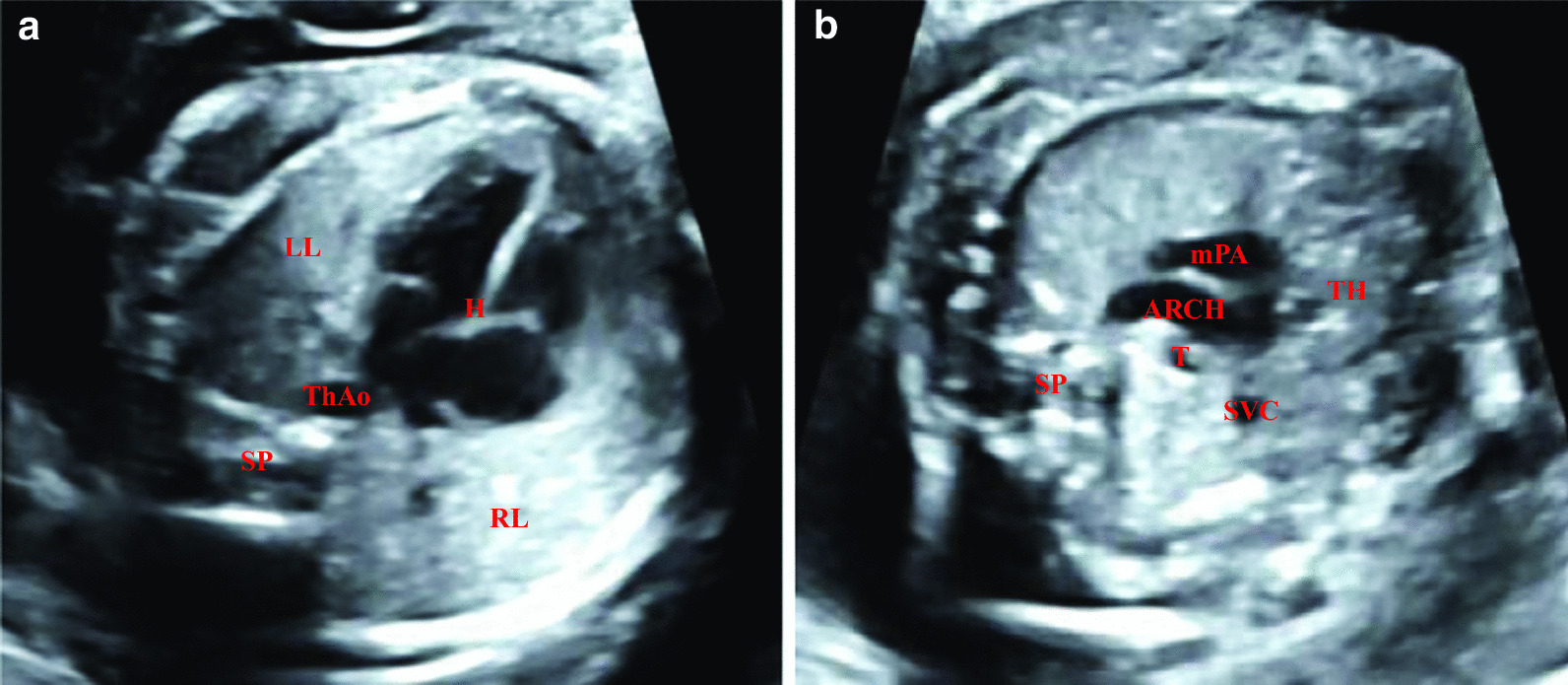
Normal ultrasonography of fetal thorax. **a** normal ultrasonography of fetal heart four-chamber view section; **b** normal ultrasonography of fetal three vessels trachea view section. H: heart, ThAO: thoracic aorta, SP: spine, LL: left lung, RL: right lung, SVC: superior vena cava, mPA: main pulmonary artery, ARCH: aortic arch, T: trachea, TH: thymus

The fetal thorax is mainly composed of skeletal thoracic frame, which is quasi-circular, and thoracic internal organs, including the heart, large blood vessels, lungs, trachea and thymus. The myocardium and lungs are moderately echogenic, and the cardiac chamber is echoless on four-chamber view section. The three vessels and trachea view section shows that the large vessel wall and tracheal wall are high echo, the lumen are echoless and the thymus is medium–low echo (Fig. 2).

### Normal reference range of fetal thoracic measurements

**Fig. 3:**
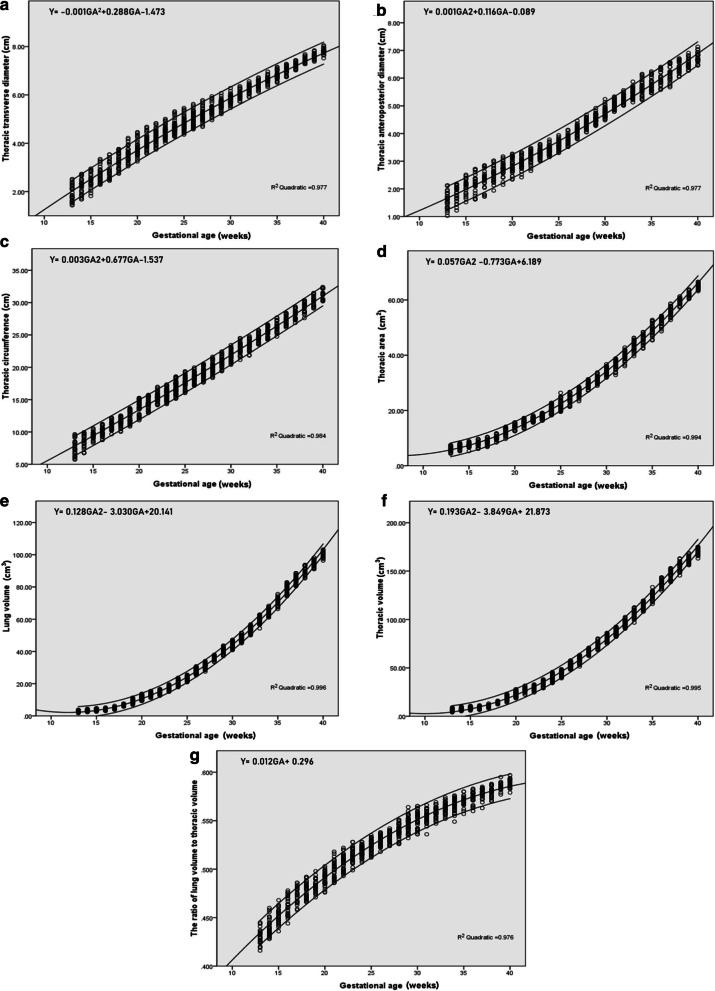
Scatterplot of fetal thoracic parameters and gestational age. **a** Thoracic transverse diameter; **b** Thoracic anteroposterior diameter; **c** Thoracic circumference; **d** Thoracic area; **e** Lung volume; **f** Thoracic volume; **g** Lung-to-thoracic volume ratio. The three curves show the 2.5th centile, mean and 97.5th centile, respectively

Correlation between fetal thoracic transverse diameter, anteroposterior diameter, thoracic circumference, thoracic area, lung volume, thoracic volume, lung-to-thoracic volume ratio and GA had high significance (*P* < 0.0001 respectively). Additionally, all fetal thoracic indicators increased with GA using a quadratic regression equation. Tables 1 and 2 show the nomograms of thoracic 2D and 3D measurements for each gestational week. Figure 3 represents the correlation and scatterplot of fetal thoracic parameters and GA.

**Table 1:** Nomograms of 2D fetal thoracic parameters from 13 to 40 gestational week (n = 1077)

GA(No.)	Thoracic transverse diameter (cm)					Thoracic anteroposterior diameter (cm)					Thoracic circumference (cm)					Thoracic area (cm^2^)				
	*P*2.5	*P*50	*P*97.5	$\overline{x}$	*SD*	*P*2.5	*P*50	*P*97.5	$\overline{x}$	*SD*	*P*2.5	*P*50	*P*97.5	$\overline{x}$	*SD*	*P*2.5	*P*50	*P*97.5	$\overline{x}$	*SD*
13(28)	1.43	1.85	2.52	1.93	0.33	1.03	1.58	2.13	1.58	0.28	5.73	7.43	9.64	7.62	1.25	4.37	5.97	7.52	6.01	0.90
14(30)	1.68	2.27	2.73	2.22	0.31	1.22	1.86	2.18	1.82	0.26	6.73	8.68	9.95	8.62	0.96	5.26	7.15	9.32	6.92	0.81
15(27)	1.82	2.49	2.95	2.46	0.29	1.52	2.14	2.51	2.09	0.25	8.31	9.48	10.56	9.46	0.56	5.31	7.51	8.89	7.51	0.74
16(29)	2.28	2.65	3.35	2.70	0.29	1.62	2.26	2.72	2.21	0.31	8.75	10.22	11.23	10.09	0.69	6.12	7.91	9.71	7.92	1.07
17(26)	2.45	2.86	3.54	2.92	0.34	1.72	2.40	2.85	2.36	0.31	9.65	11.00	12.21	11.04	0.68	6.79	8.80	10.43	8.78	1.05
18(34)	2.74	3.28	3.76	3.29	0.30	1.94	2.58	3.02	2.54	0.25	10.04	11.61	12.53	11.55	0.65	8.42	10.25	11.74	10.32	0.76
19(34)	3.05	3.55	4.22	3.57	0.29	2.15	2.75	3.17	2.74	0.22	10.85	12.59	10.86	12.43	0.86	9.73	12.61	14.10	12.41	1.12
20(36)	3.25	3.76	4.46	3.79	0.30	2.34	2.89	3.27	2.87	0.26	11.81	14.05	15.26	13.92	1.02	12.52	14.08	15.69	14.15	0.88
21(40)	3.57	4.07	4.68	4.08	0.26	2.42	2.93	3.32	2.94	0.20	12.94	14.37	15.75	14.46	0.76	13.42	15.71	17.25	15.65	0.99
22(47)	3.75	4.26	4.83	4.27	0.25	2.67	3.18	3.47	3.16	0.18	14.17	15.40	16.95	15.37	0.67	15.64	16.79	18.17	16.86	0.62
23(59)	4.06	4.47	4.98	4.46	0.22	3.02	3.27	3.61	3.28	0.14	14.38	15.72	17.28	15.81	0.75	16.36	17.68	19.11	17.76	0.78
24(62)	4.28	4.61	5.29	4.64	0.22	3.16	3.39	3.74	3.40	0.13	15.21	16.74	18.54	16.86	0.88	18.39	20.34	22.72	20.24	1.25
25(60)	4.39	4.78	5.43	4.81	0.23	3.31	3.58	3.91	3.60	0.13	16.09	17.64	19.31	17.73	0.75	19.60	22.28	25.70	22.25	1.48
26(55)	4.60	5.02	5.60	5.02	0.26	3.52	3.76	4.13	3.78	0.16	17.20	18.52	19.87	18.51	0.63	21.40	24.37	26.68	24.39	1.36
27(49)	4.70	5.12	5.77	5.17	0.23	3.81	4.13	4.44	4.11	0.15	17.68	19.17	20.55	19.18	0.74	23.54	26.47	28.48	26.47	1.03
28(47)	4.97	5.36	5.93	5.40	0.19	4.05	4.36	4.66	4.35	0.15	18.40	19.95	21.58	19.97	0.77	27.25	29.34	31.26	29.25	1.02
29(44)	5.22	5.62	6.06	5.61	0.17	4.28	4.55	4.91	4.54	0.15	19.41	20.86	22.41	20.89	0.70	29.01	31.64	33.70	31.50	1.06
30(43)	5.48	5.82	6.27	5.82	0.16	4.48	4.75	5.23	4.76	0.17	20.41	21.92	23.40	21.87	0.75	31.92	33.83	36.70	34.07	1.25
31(42)	5.73	6.04	6.36	6.03	0.15	4.79	5.06	5.33	5.06	0.15	21.19	22.62	24.51	22.78	0.80	32.90	36.93	38.83	36.67	1.42
32(41)	5.92	6.21	6.56	6.23	0.15	5.01	5.26	5.56	5.24	0.14	21.84	23.53	24.82	23.51	0.75	36.06	39.76	42.72	39.83	1.49
33(37)	6.13	6.42	6.72	6.43	0.14	5.13	5.47	5.87	5.45	0.17	23.51	24.51	25.71	24.54	0.67	39.67	43.62	46.28	43.42	1.66
34(35)	6.32	6.62	6.96	6.63	0.14	5.21	5.62	6.15	5.64	0.24	24.27	25.42	26.61	25.43	0.68	42.38	46.27	48.36	45.99	1.62
35(32)	6.51	6.81	7.16	6.82	0.17	5.46	5.74	6.24	5.79	0.24	24.75	26.27	27.68	26.25	0.72	45.38	49.23	52.76	49.27	1.87
36(31)	6.74	6.97	7.28	7.00	0.15	5.58	5.92	6.40	5.98	0.25	26.18	27.30	28.42	27.27	0.65	48.63	52.61	54.24	52.23	1.40
37(29)	7.02	7.21	7.43	7.23	0.12	5.67	6.18	6.58	6.18	0.27	26.80	28.34	29.41	28.24	0.69	52.73	56.71	58.62	56.23	1.48
38(27)	7.28	7.43	7.68	7.44	0.11	6.05	6.35	6.71	6.38	0.20	27.61	29.42	30.38	29.23	0.74	57.31	59.46	61.28	59.34	1.06
38(27)	7.38	7.61	7.84	7.61	0.12	6.27	6.49	6.91	6.54	0.18	29.11	30.18	31.48	30.11	0.71	60.35	61.59	63.81	61.87	0.91
40(26)	7.52	7.83	8.05	6.48	0.12	6.46	6.78	7.13	6.76	0.18	30.21	31.32	32.41	31.32	0.71	63.18	64.82	66.54	65.95	0.99

*GA* gestational age, *SD* standard deviation

**Table 2:** Nomograms of 3D fetal thoracic parameters from 13 to 40 gestational week (n = 1077)

GA(No.)	Lung volume (cm^3^)					Thoracic volume (cm^3^)					Lung-to-thoracic volume ratio				
	*P*2.5	*P*50	*P*97.5	$\overline{x}$	*SD*	*P*2.5	*P*50	*P*97.5	$\overline{x}$	*SD*	*P*2.5	*P*50	*P*97.5	$\overline{x}$	*SD*
13(28)	1.75	2.72	3.85	2.79	0.61	4.03	6.24	9.04	6.50	1.43	0.416	0.430	0.445	0.430	0.008
14(30)	2.36	3.12	4.32	3.18	0.55	5.25	7.06	10.08	7.22	1.27	0.428	0.439	0.460	0.441	0.009
15(27)	2.64	3.67	4.74	3.72	0.54	5.91	8.15	10.69	8.26	1.17	0.436	0.451	0.468	0.451	0.008
16(29)	3.12	3.88	5.23	4.04	0.53	6.64	8.52	11.26	8.74	1.17	0.444	0.461	0.478	0.462	0.010
17(26)	3.83	4.81	6.25	4.82	0.57	8.24	10.08	13.04	10.18	1.18	0.458	0.474	0.486	0.473	0.008
18(34)	5.84	6.72	7.64	6.73	0.51	12.34	14.01	15.93	14.04	1.01	0.466	0.480	0.492	0.479	0.007
19(34)	6.83	8.83	11.34	9.13	1.11	14.25	18.20	23.15	18.90	2.32	0.471	0.483	0.498	0.483	0.006
20(36)	9.28	11.43	13.72	11.38	1.23	18.75	23.64	27.94	23.22	2.51	0.476	0.491	0.490	0.007	0.007
21(40)	11.16	13.59	15.49	13.54	1.07	22.32	27.04	31.54	27.10	2.16	0.489	0.500	0.518	0.500	0.008
22(47)	12.63	14.84	16.61	14.78	0.96	24.88	28.90	32.79	29.08	1.89	0.493	0.510	0.522	0.508	0.007
23(59)	15.07	17.37	19.38	17.29	1.34	29.08	33.86	37.76	33.65	2.61	0.501	0.514	0.528	0.514	0.005
24(62)	17.91	20.26	23.46	20.37	1.50	34.51	39.21	45.08	39.27	2.86	0.508	0.520	0.528	0.519	0.005
25(60)	21.03	23.41	25.49	23.41	1.24	39.78	44.87	48.49	44.63	2.40	0.513	0.525	0.534	0.525	0.005
26(55)	25.40	28.23	30.69	28.01	1.46	48.17	53.27	57.40	52.90	2.57	0.520	0.529	0.538	0.529	0.005
27(49)	27.76	31.13	33.96	30.96	1.44	51.93	58.36	63.23	58.01	2.72	0.524	0.535	0.542	0.534	0.005
28(47)	33.47	35.76	37.87	35.97	1.07	62.61	66.71	70.67	66.67	2.02	0.526	0.539	0.551	0.540	0.006
29(44)	37.18	40.40	43.59	40.43	1.53	68.52	74.16	80.26	74.02	2.60	0.532	0.548	0.564	0.546	0.007
30(43)	40.90	44.16	47.21	44.28	1.70	75.31	80.36	86.11	80.27	2.79	0.536	0.553	0.565	0.552	0.008
31(42)	45.15	48.38	51.52	48.33	1.85	82.21	87.35	92.52	87.09	3.00	0.537	0.555	0.566	0.555	0.006
32(41)	50.06	53.71	56.79	53.42	1.83	90.67	95.55	102.55	95.62	3.24	0.547	0.559	0.570	0.559	0.005
33(37)	54.33	59.72	62.85	59.53	2.07	96.84	105.50	112.56	105.70	3.99	0.552	0.563	0.574	0.563	0.005
34(35)	61.17	64.71	68.34	64.67	1.96	108.27	114.50	119.42	114.23	3.33	0.549	0.566	0.576	0.566	0.006
35(32)	66.38	71.49	75.62	71.17	2.11	116.17	125.30	133.37	124.85	3.88	0.557	0.570	0.580	0.570	0.005
36(31)	74.18	79.38	82.95	79.02	2.49	129.98	138.51	143.51	137.63	4.01	0.560	0.574	0.583	0.574	0.006
37(29)	80.42	84.62	88.64	84.69	2.40	138.66	146.40	153.01	146.53	4.33	0.563	0.579	0.585	0.578	0.005
38(27)	85.39	90.73	93.73	90.11	2.50	145.47	156.67	163.10	155.28	4.40	0.570	0.581	0.588	0.580	0.005
38(27)	91.03	94.84	98.21	94.74	1.96	156.14	162.09	168.53	161.82	3.44	0.577	0.585	0.595	0.585	0.003
40(26)	96.38	100.46	103.28	100.23	1.80	163.08	170.74	175.05	170.21	3.26	0.579	0.590	0.597	0.589	0.004

*GA* gestational age, *SD* standard deviation

### Comparison of thoracic parameters between abnormal groups and normal group

The comparison of fetal thoracic parameters between the abnormal groups (SD group, CDH group, PS group and CCAM group) and the normal group is shown in Table 3. All the thoracic parameters in SD group were significantly lower than those in normal group (*P* < 0.0001). Similarly, the lung-to-thoracic volume ratio in CDH, PS and CCAM group were lower and had statistical significance (*P* < 0.0001). Compared with normal group, the lung volume in CDH, PS and CCAM group were lower (*P* < 0.05). However, all the 2D parameters and thoracic volume had no statistical differences between CDH, PS, CCAM group and normal group (*P* > 0.05).

**Table 3:** The comparison of fetal thoracic measurements between abnormal groups and normal group

Group (n)	GA (weeks)	Thoracic transverse diameter (cm)	Thoracic anteroposterior diameter (cm)	Thoracic circumference (cm)	Thoracic area (cm^2^)	Lung volume (cm^3^)	Thoracic volume (cm^3^)	Lung-to-thoracic volume ratio
Normal group (n = 1077)	26.35 ± 7.19	5.04 ± 1.54	4.06 ± 1.41	18.91 ± 6.21	28.24 ± 16.46	35.55 ± 27.81	64.27 ± 47.10	0.526 ± 0.041
SD group (n = 15)	19.89 ± 3.74^*^	2.56 ± 0.78^*^	1.92 ± 0.49^*^	9.77 ± 3.46^*^	10.14 ± 5.14^*^	7.34 ± 5.14^*^	15.46 ± 10.04^*^	0.454 ± 0.039^*^
CDH group (n = 31)	25.06 ± 4.07	4.83 ± 0.83	3.73 ± 0.0.88	17.32 ± 3.42	23.50 ± 8.1	21.93 ± 13.19^#^	56.82 ± 28.69	0.370 ± 0.035^*^
PS group (n = 25)	25.42 ± 4.85	4.87 ± 1.10	3.92 ± 1.02	18.60 ± 4.22	25.49 ± 11.19	24.03 ± 29.87^#^	54.31 ± 29.87	0.411 ± 0.063^*^
CCAM group (n = 36)	25.84 ± 4.91	4.78 ± 1.08	3.82 ± 1.00	18.03 ± 4.14	24.41 ± 10.67	23.98 ± 16.04^#^	52.31 ± 29.62	0.427 ± 0.064^*^

*GA* gestational age, *SD* skeletal dysplasia, *CDH* congenital diaphragmatic hernia, *PS* pulmonary sequestration, *CCAM* congenital cystic adenomatoid malformation

^*^P < 0.0001 versus normal group

^#^*P* < 0.05 versus normal group

### Intra-observer and inter-observer reliability and agreement

**Fig. 4:**
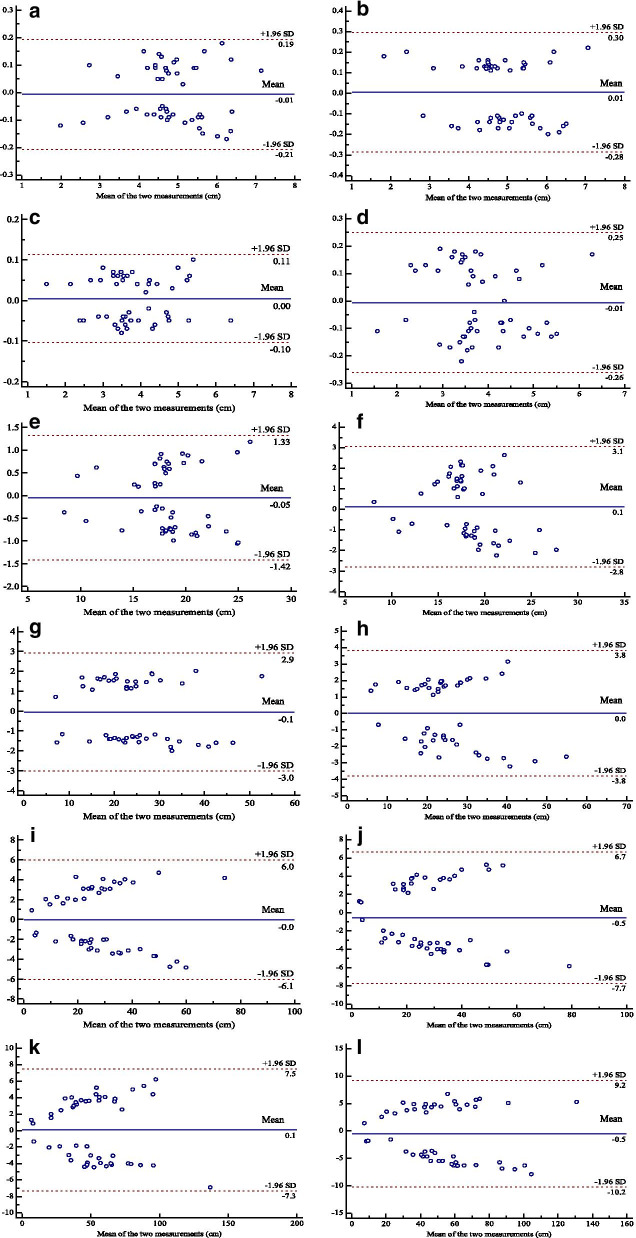
Bland–Altman plots of intra-observer and inter-observer agreement of thoracic measurements. **a**, **b** Thoracic transverse diameter; **c**, **d** Thoracic anteroposterior diameter; **e**, **f** Thoracic circumference; **g**, **h** Thoracic area; **i**, **j** Lung volume; **k**, **l** Thoracic volume. The blue solid curve represents the mean difference, while the red dashed curves show the 95% LoA

The intra- and inter-observer reliability and agreement of fetal thoracic measurements were excellent (ICC > 0.90 and narrow 95% LoA respectively), which are shown in Table 4 and Fig. 4. Among them, the intra-observer reliability and agreement were the best in measuring the fetal thoracic anteroposterior diameter, with ICC = 0.9992, 95% confidence interval (95%CI) 0.9986–0.9995 and the mean difference was 0.0048 cm (95% LoA: − 0.1039–0.1135).

**Table 4:** Intra-observer and inter-observer reliability and agreement

Parameter	Intra-observer		Inter-observer	
	ICC (95%CI)	Mean difference (95% LoA)	ICC (95%CI)	Mean difference (95% LoA)
Thoracic transverse diameter	0.9981 0.9967–0.9989	0.0062 − 0.2070–0.1946	0.9899 0.9823–0.9942	0.0054 − 0.2845–0.2953
Thoracic anteroposterior diameter	0.9992 0.9986–0.9995	0.0048 − 0.1039–0.1135	0.9948 0.9908–0.9970	− 0.0060 − 0.2613–0.2493
Thoracic circumference	0.9808 0.9665–0.9890	− 0.0470 − 0.2463–0.1523	0.9203 0.8662–0.9531	0.1286 − 2.7965–3.0537
Thoracic area	0.9874 0.9781–0.9928	− 0.0556 − 0.4865–0.3753	0.9798 0.9653–0.9883	0.0218 − 3.8034–3.8470
Lung volume	0.9388 0.9631–0.9878	− 0.0428 − 0.9143–0.8287	0.9191 0.9466–0.9823	− 0.5338 − 7.7247–6.6571
Thoracic volume	0.9590 0.9807–0.9937	0.0922 − 0.9856–1.1700	0.9411 0.9672–0.9892	− 0.4964 − 10.2373–9.2445

*ICC* intraclass correlation coefficient, *CI* confidence interval, *LoA* limits of agreement

## Discussion

Various ultrasound investigations have focused on the application of ultrasound measurements to predict the fetal lung development. For example, Triebwasser et al. [3] used lung area to prenatally diagnose PH and found that the sensitivity, specificity, and both positive and negative predictive values were all more than 75%. Miric et al. [6] stated that fetal volume was critical in early detecting of PH. Moreover, Britto et al. [11] proposed that there were a high correlation between the 2D and 3D ultrasound in the evaluation of fetal lung volume. In terms of the fetal thorax, some studies mentioned the usefulness of fetal thoracic area [12], thoracic circumference [2, 3] and thoracic volume [6] in prenatal diagnosis, but few systematic studies establish the fetal thoracic nomogram and assess the application in diagnosing thoracic malformations. Ultrasound could diagnose CTM such as congenital pleural effusion, CHD and bronchopulmonary sequestration before 16 gestational weeks, which is beneficial for prenatal counseling and making early decisions concerning deadly fetal malformations [5]. Suyama et al. [12] measured thoracic area and used lung-to-thorax transverse area ratio to confirm the lung size after thoracoamniotic shunting, and concluded that the area ratio was connected with the prognosis of fetal primary hydrothorax. Research indicated that the area ratio of liver herniation and thorax was essential for the evaluation of severe degree of liver herniation in CDH individuals [13]. In terms of thoracic volume measuring method, Miric Tesanic et al. [6] demonstrated that both lung volumes plusing heart volume was thoracic volume. However, this is not completely accurate because they disregarded other organs’ volumes in the mediastinum, like the thymus. Moreover, they used the 3D multiplane reconstruction mode to measure the fetal lung and heart volume by adding different slices together from the diaphragm to the clavicle. Compared with VOCAL technique, it is difficult to calculate the lower lung volume although there is a similar volume result between multiplane and VOCAL technique [7, 8]. In addition, 3D multiplane reconstruction method is a cumbersome and time-consuming procedure, especially for inexperienced physicians, which limits its clinical application. VOCAL technique is the most popular method for volume measuring because it is convenient, time-efficient, cost-effective and its reliability and agreement are both high [14–17]. Furthermore, our study shows high reliability with all ICC > 0.90 and excellent agreement with narrow 95% LoA, respectively [9, 10]. VOCAL technique can be used to measure regular organs such as bladder and irregular organs such as lung and thymus. In addition, the organ contour in each rotation section can be modified, which makes the volume more accurate. Finally, most previous studies used the VOCAL technique with rotation angle of 30° [7, 14, 16, 18, 19], we chose the rotation angle of 15° to make the volume more precise.

Consequently, we propose to use 2D ultrasound and 3D ultrasound VOCAL technique to measure fetal thoracic 2D parameters and 3D volumes and establish the reference range for all fetal thoracic parameters. This study results demonstrate that both the 2D and the 3D thoracic parameters increase with the GA. Moreover, the associations between the each thoracic indicator and the GA are high and best illustrated by quadratic equations. Thus, thoracic transverse and anteroposterior diameter, thoracic circumference, thoracic area, lung volume, thoracic volume and lung-to-thorax volume ratio can be treated as new biometric parameters, which are practical to evaluate the development of fetal thorax.

We hypothesize that 2D thoracic parameters can be used to preliminarily assess the basic condition of the fetal thorax, while 3D thoracic parameters further evaluate the fetal lung and thorax, which is beneficial for CTM diagnosis. Our study results also verify that the thoracic 2D measurements in skeletal dysplasia (SD) group were significantly lower than those in normal group, indicating that SD greatly influences the 2D parameters, and can be diagnosed by 2D ultrasound. Furthermore, both lung volume and thoracic volume were much smaller than the volumes of normal group. This might be because the SD fetus has a narrow skeletal thorax [20, 21] and causes the significant diminish of thoracic volume, which results in limited development of the fetal lung and more significant volume reduction. Thus, the lung-to-thorax volume ratio of the SD fetus is decreased markedly compared with the normal group. However, the 2D fetal thoracic parameters in CDH, PS and CCAM group are all within the reference range, showing that it is not statistically significant to measure fetal thoracic 2D parameters to diagnose those deformities. On the other hand, there is a statistical difference of lung volume and lung-to-thorax volume ratio between the case groups and normal group. For CDH group, due to the diaphragm defect, the abdominal contents herniate into the fetal thorax [13, 22], which squeezes the lung tissue and causes the restricted lung development, even results in pulmonary dysplasia. Although the thoracic volume of the CDH fetus did not have statistical difference from the normal fetus, it showed a trend to a lower value. The low case number might be one potential reason, as such, we need to increase the CDH sample cases in the future study to confirm whether the CHD fetal thoracic volume is really lower than normal fetus. Because of the lung volume decrease and non-obvious thoracic volume change, the lung-to-thorax volume ratio is significantly diminished. Likewise, the lung volumes of the PS fetus and CCAM fetus are also reduced, the reason might be that PS and CCAM are both congenital pulmonary malformations, PS is non-functional sequestered lung tissue which receives blood supply from the circulating arteries [5, 23], meanwhile, CCAM is characterized by abnormal bronchial airway hyperplasia and lack of normal alveoli [5, 24]. Both conditions affect the normal progress of the fetal lung and bring about lower lung volume. Conversely, the abnormal lung mass of PS and CCAM does not affect the development of fetal skeletal thorax and diaphragm, so the difference of thoracic volume between the PS, CCAM group and normal group is not significant. As a result, the lung-to-thorax volume ratio of PS and CCAM fetuses is significantly reduced.

Compared with previous studies [19, 25], our research has a large sample size including 1077 normal fetuses from 13 gestational weeks to 40 gestational weeks, which makes the reference data more representative and reliable. Moreover, it enriches the normal fetal biostatistics and helps clinicians to evaluate and follow up fetal development comprehensively. Secondly, our study, including both 2D and 3D thoracic parameters, is the first research project to systematically evaluate the development of fetal thorax. This is meaningful and practical to comprehensively distinguish the normal and pathological fetal thoracic state [19]. In addition, we find that the 2D fetal thoracic parameters can be used to initially evaluate the fetal thoracic development and diagnose skeletal thoracic deformity. In the meantime, the lung volume, thoracic volume and lung-to-thorax volume ratio that reconstructed by 3D VOCAL technique, are useful to diagnose and differentiate CDH fetus, PS fetus and CCAM fetus. Combination of 2D and 3D ultrasound VOCAL technique can guide doctors to carry out early and appropriate measurements of fetuses with thoracic malformations.

Limitations of this study: firstly, the 3D ultrasound VOCAL technique is susceptible to fetal position, amniotic fluid volume or obese pregnant women. Secondly, it is difficult to clearly identify the inferior boundary of fetal lung on some rotation planes, since it is easily affected by the attenuation of the fetal ossific rib or spine, especially in the third trimester of pregnancy. This might reduce the accuracy of volume measurement.

## Conclusion

We establish an integrated nomograms of fetal thoracic transverse and anteroposterior diameter, thoracic circumference, thoracic area, lung volume, thoracic volume and lung-to-thorax-volume ratio by 2D and 3D ultrasound. All thoracic measurements have high intra- and inter-observer reliability and agreement and increase with the GA, the correlation between each measurement and GA is excellent. Meanwhile, we find that combining 2D ultrasound with 3D VOCAL technique has a high value in diagnosing CTM.

## Data Availability

The datasets used and/or analyzed during the current study are available from the corresponding author on reasonable request.

## Abbreviations

CTM: Congenital thoracic malformations
SD: Skeletal dysplasia
CDH: Congenital diaphragmatic hernia
PS: Pulmonary sequestration
CCAM: Congenital cystic adenomatoid malformation
PH: Pulmonary hypoplasia
2D: Two-dimensional
3D: Three-dimensional
VOCAL: Virtual Organ Computer-aided Analysis
GA: Gestational age
SD: Standard deviation
ICC: Intraclass correlation coefficient
LoA: Limits of agreement
